# Immune cells differentiation in osteoarthritic cartilage damage: friends or foes?

**DOI:** 10.3389/fimmu.2025.1545284

**Published:** 2025-03-25

**Authors:** Mingxiang Liu, Chaoqun Wu, Chaofan Wu, Zulong Zhou, Run Fang, Chenfeng Liu, Rende Ning

**Affiliations:** Department of Orthopedics, The Third Affiliated Hospital of Anhui Medical University (The First People’s Hospital of Hefei), School of Life Science, Anhui Medical University, Hefei, Anhui, China

**Keywords:** osteoarthritis, immune cell, cartilage damage, inflammation, immune therapy

## Abstract

Osteoarthritis (OA) is a chronic disease primarily characterized by degenerative changes in articular cartilage and synovitis, for which there are currently no targeted or curative therapies available in clinical practice. In recent years, the in-depth analysis of OA using single-cell sequencing and immunomics technologies has revealed the presence of multiple immune cell subsets, as well as different differentiation states within the same subset, in OA. Through immune-immune and immune-joint tissue interactions, these cells collectively promote or inhibit the progression of arthritis. This complex immune network, where “friends and foes coexist,” has made targeted therapeutic strategies aimed at directly eliminating immune cells challenging, highlighting the urgent need for a detailed review of the composition, distribution, functional heterogeneity, therapeutic potential, and potential risks of immune subsets within the joint. Additionally, the similarities and differences between OA and rheumatoid arthritis (RA) in terms of diagnosis and immunotherapy need to be precisely understood, in order to draw lessons from or reject RA-based immunotherapies. To this end, this review summarizes the major triggers of inflammation in OA, the differentiation characteristics of key immune cell subsets, and compares the similarities and differences between OA and RA in diagnosis and treatment. It also outlines the current immunomodulatory strategies for OA and their limitations. Furthermore, we provide a detailed and focused discussion on immune cells that act as “friends or foes” in arthritis, covering the M1/M2 polarization of macrophages, functional heterogeneity of neutrophils, unique roles of dendritic cells at different maturation states, the balance between pro-inflammatory T cells and regulatory T cells (Tregs), and the diverse functions of B cells, plasma cells, and regulatory B cells (Bregs) in OA. By interpreting the roles of these immune cells, this review clarifies the dynamic changes and interactions of immune cells in OA joints, providing a theoretical foundation for more precise targeted interventions in future clinical practice.

## 1 Introduction

Osteoarthritis (OA) is one of the most common forms of arthritis, affecting over 500 million people worldwide, and damage to articular cartilage is one of the most prevalent pathological changes in OA (1–4). Risk factors for OA include obesity, female sex, aging, knee injury, and immune dysregulation (5). The primary symptoms of OA are knee pain, swelling, and limited mobility, with pain caused by cartilage damage and osteophyte formation being the most significant issue affecting patients’ daily lives (6). Although OA has traditionally been considered a mechanical wear-and-tear disease, recent advances in single-cell sequencing and immunomics have unveiled the heterogeneity of immune cell subsets and their dynamic roles in OA progression, revealing a dualistic nature where immune cells can act as both “friends” and “foes” in the joint microenvironment (7–9).

Articular cartilage is an avascular, aneural, and alymphatic tissue (10), typically composed of 95% extracellular matrix (ECM) and 3-5% chondrocytes (11, 12). The three-dimensional microstructure of the ECM is complex, containing structural proteins such as collagen (primarily type II collagen (Col II)), fibronectin, laminin, glycosaminoglycans (GAGs), and bioactive growth factors (11). Although cartilage tissue lacks blood vessels and nerves, during the progression of OA, immune cells infiltrate the joint cavity and cartilage microenvironment through various pathways, participating in the regulation of inflammatory responses and tissue repair.

However, the role of immune cells in cartilage damage during OA remains controversial in existing research, with debates over whether they act as “foes” or “friends”. Some studies suggest that immune cells can both aid in clearing damage and repairing tissues during the development of OA, while also potentially overreacting and causing further cartilage damage (10, 13, 14). On one hand, immune cells directly or indirectly contribute to cartilage destruction by releasing pro-inflammatory factors (such as TNF-α, IL-1β, and IL-6) and matrix-degrading enzymes (such as MMPs and ADAMTS) (15). On the other hand, certain immune cell subsets may delay OA progression through anti-inflammatory and tissue repair mechanisms. For example, regulatory T cells, regulatory B cells, and M2 macrophages can secrete anti-inflammatory factors(such as IL-10,TGF-β) and chondrogenic cytokines (such as TGF-β, IGF-1) to promote chondrocyte proliferation and cartilage repair (16–18). Therefore, an in-depth exploration of the dual roles of immune cell differentiation in OA cartilage damage not only helps to reveal the complex pathogenesis of OA but also provides a critical foundation for developing novel immune-based therapeutic strategies.

Furthermore, the similarities and differences between OA and rheumatoid arthritis (RA) in terms of immune mechanisms and therapeutic strategies warrant careful consideration. While RA is a classic autoimmune disease driven by systemic inflammation (19), OA is primarily a localized degenerative condition with low-grade inflammation. However, emerging evidence suggests overlapping immune pathways between the two diseases, raising questions about the applicability of RA-targeted immunotherapies in OA. For example, biologics targeting TNF or IL-6 have shown remarkable efficacy in RA but limited success in OA (20), underscoring the need for OA-specific therapeutic approaches.

In summary, the immune system acts as a double-edged sword in the process of cartilage damage during osteoarthritis. This review aims to provide a comprehensive overview of the immune cell landscape in OA, focusing on the differentiation and functional heterogeneity of key immune cell subsets, including macrophages, neutrophils, dendritic cells, T cells, and B cells. We will discuss their dual roles in OA progression, highlighting the delicate balance between pro-inflammatory and anti-inflammatory responses. Additionally, we will compare the immunological features and therapeutic strategies of OA and RA, emphasizing the lessons that can be learned from RA immunotherapy while avoiding its pitfalls. Finally, we will summarize current immunomodulatory strategies for OA, their limitations, and future directions for developing precise and effective therapies. By elucidating the dynamic interactions and functional plasticity of immune cells in OA, this review aims to provide a theoretical foundation for advancing targeted interventions in OA management.

## 2 Triggers of immune system abnormalities in OA

The abnormal activation of the immune system in osteoarthritis (OA) is a dynamic process driven by the synergistic effects of multiple factors. Mechanical stress, as a core physical trigger, directly damages chondrocytes and the extracellular matrix, leading to the release of damage-associated molecular patterns (DAMPs, such as high-mobility group box 1 (HMGB1) and fibronectin fragments). These DAMPs activate the TLR/NF-κB/NLRP3 inflammasome pathway in synovial macrophages and dendritic cells, initiating innate immune responses (21–23). Simultaneously, aging-related metabolic disturbances (such as mitochondrial dysfunction, reactive oxygen species (ROS) accumulation, and lipid metabolism abnormalities) reshape the immune microenvironment through oxidative stress and the senescence-associated secretory phenotype (SASP) (24), suppressing regulatory T cell (Treg) function and enhancing Th17-mediated pro-inflammatory responses, thereby establishing a chronic low-grade inflammatory state (25–27).

Notably, mechanical stress and metabolic imbalances exhibit synergistic and mutually reinforcing effects. On one hand, mechanical stress directly participates in the pathological progression of osteoarthritis (OA) by influencing chondrocyte metabolism and inflammatory responses. For example, appropriate mechanical stress can activate the TGF-β1 signaling pathway, promoting anabolic metabolism in chondrocytes and secretion of extracellular matrix (ECM) to maintain cartilage integrity (28). However, excessive mechanical stress exacerbates the inflammatory response and pyroptosis of chondrocytes through inflammatory signaling pathways such as NF-κB (29). On the other hand, metabolic imbalances, such as obesity and diabetes, alter the immune-metabolic environment within the joint, increasing levels of pro-inflammatory factors and further aggravating cartilage damage (30, 31). These metabolic disturbances also lead to mitochondrial dysfunction in chondrocytes, impairing their energy metabolism and antioxidant capacity (32). Mechanistically, increased joint load in obese patients, coupled with elevated levels of adipokines (such as leptin), promotes macrophage infiltration. Additionally, mitochondrial autophagy defects in senescent chondrocytes amplify the release of DAMPs induced by mechanical damage (33), further recruiting inflammatory monocytes and creating a “metabolic-mechanical-immune” cascade (34–36). The cross-interaction of these triggering factors reveals the complex mechanisms underlying the transition from local biomechanical imbalance to systemic immune dysregulation in OA, providing a critical theoretical foundation for targeted interventions (37, 38).

## 3 Differentiation of immune cells in OA

Previous studies have attributed the pathogenesis of osteoarthritis (OA) to cartilage wear and tear. However, recent research suggests that OA is actually a chronic inflammatory condition involving extensive participation of immune cells (39). With in-depth exploration of the pathogenesis of OA, scholars now believe that the disease is driven by early innate immune responses, which gradually catalyze degenerative changes and ultimately lead to alterations in the joint microenvironment.

During the onset of OA, various immune cells and cytokines are key factors influencing OA repair. For instance, macrophages differentiate into different subtypes, participate in synovial inflammatory responses, and exacerbate cartilage damage by releasing inflammatory mediators (such as IL-1β,TNF-α) and proteases (such as MMPs,ADAMTs) (16, 22, 40). Natural killer (NK) cells are also involved, causing certain damage to joint tissues. T cells differentiate into various subsets, such as T helper 1 (Th1), T helper 2 (Th2), and T helper 17 (Th17) cells, which participate in immune regulation and inflammatory responses in OA by secreting different cytokines (41). Among these, the inflammatory factors secreted by Th1 and Th17 cells promote cartilage degradation and OA progression.

At the same time, the differentiation of immune cells is influenced by the joint microenvironment. In OA, changes in the joint microenvironment affect the differentiation and function of immune cells, thereby exacerbating the disease. For example, inflammatory factors and chemokines in synovial fluid can attract and activate immune cells, promoting their differentiation and proliferation, which intensifies inflammatory responses and cartilage damage (42). On the other hand, immune cells and their differentiation products also play a role in immune regulation, helping to maintain joint immune homeostasis (43). For instance, M2 macrophages, regulatory B cells, and regulatory T cells can secrete anti-inflammatory factors (such as IL-10,TGF-β), suppressing inflammatory responses and cartilage damage (7, 17); Some immune cells can also promote chondrocyte proliferation and repair, aiding in the regeneration and recovery of joint tissues.

Therefore, the differentiation of immune cells in OA has both damaging and protective effects, acting as a double-edged sword. In-depth research into the differentiation of immune cells and their mechanisms in OA will help us better understand the pathological processes of OA and provide new insights and approaches for its prevention and treatment.

## 4 Immunological differentiation between osteoarthritis and rheumatoid arthritis

Osteoarthritis (OA) and rheumatoid arthritis (RA) are both diseases involving immune factors. Although their pathogenesis differs, they share similarities in multiple aspects, making it essential to distinguish between the two clinically and apply targeted treatments. Currently, the clinical methods for differentiating osteoarthritis (OA) from rheumatoid arthritis (RA) include: (1) serological markers, such as the presence of rheumatoid factor (RF) and anti-cyclic citrullinated peptide antibodies (anti-CCP antibodies) in RA; (2) X-ray: OA is characterized by asymmetric joint space narrowing, while RA shows symmetric joint space narrowing (44, 45); (3) Magnetic Resonance Imaging (MRI): OA typically manifests as cartilage thinning, subchondral bone marrow lesions, and osteophyte formation, whereas RA shows prominent synovitis, bone marrow edema, and bone erosion (44, 45). However, OA and RA share many similarities, leading to difficulties in clinical diagnosis, their commonalities include: (1) Both OA and RA present with joint pain and often involve varying degrees of morning stiffness; (2) Pathological damage in both conditions includes cartilage injury and joint effusion; (3) Both exhibit significant inflammatory features and substantial immune cell infiltration in the synovium, although the subtypes and proportions of infiltrating immune cells differ markedly (34, 46).

Both OA and RA fall under the category of arthritis, which can easily lead to the use of incorrect treatment strategies in clinical practice. Clarifying the differences in etiology, pathological mechanisms, and immune-based treatment approaches between the two is a critical scientific issue in clinical settings. Etiologically, OA is a degenerative joint disease that primarily affects weight-bearing joints (such as the knees, hips, and ankles) and is commonly seen in middle-aged and elderly individuals (47). In contrast, RA is an autoimmune disease that can occur at any age and often involves small joints such as the metacarpophalangeal joints, wrists, and proximal interphalangeal joints (48). Pathologically, OA is mainly caused by wear and tear, aging, or prolonged mechanical stress on the articular cartilage, leading to cartilage degeneration and osteophyte formation (49, 50). RA, on the other hand, is primarily driven by immune cell infiltration and activation, which induce inflammation, resulting in synovitis and the destruction of articular cartilage and bone tissue (51).

The most typical difference between OA and RA lies in their immunological pathogenesis. In OA, prolonged mechanical stress and physical damage lead to the presence of Some foreign bodies, such as loose bodies, osteophytes, cartilage fragments, and inflammatory joint effusions, which trigger the activation of the immune system, including the complement system. This induces the infiltration of various innate immune cells, such as neutrophils and macrophages, and the release of large amounts of cytokines. Simultaneously, persistent cytokines activate adaptive immune responses, further exacerbating inflammation within the joint (30). In contrast, RA is more recognized as an autoimmune disease, where B cells produce autoantibodies against rheumatoid factor (RF) and cyclic citrullinated peptides (CCP). These autoantibodies form immune complexes with antigens, deposit in the joints, and induce the infiltration of other immune cells, collectively triggering inflammation and tissue damage (52). In summary, the treatment of OA primarily focuses on symptom relief, such as reducing pain, nourishing cartilage, and improving joint function (34, 53), while RA treatment involves more aggressive approaches to suppress antibody responses, control inflammation, and enhance joint function (19).

Additionally, based on the presence or absence of anti-CCP antibodies/RF positivity in the serum, RA can be classified into seronegative RA (SNRA) and seropositive RA (SPRA) (54). SNRA is a subtype of rheumatoid arthritis (RA) in which rheumatoid factor (RF) and anti-cyclic citrullinated peptide antibodies (anti-CCP) are undetectable in the blood, posing challenges for clinical diagnosis (55). In contrast, SPRA is characterized by the presence of RF and anti-CCP antibodies in the blood, exhibiting more aggressive inflammation, more severe joint damage, and more pronounced clinical phenotypes (56). Furthermore, SPRA demonstrates a more significant imbalance in T helper 17 cells (Th17)/regulatory T cells (Treg) compared to SNRA, highlighting important immunological differences between the two (57). Therefore, mainstream research has focused more on the connections and distinctions between SPRA and OA.

The study by Zhang F et al. emphasized that SPRA is characterized by plasma blast expansion, follicular helper T cell (Tfh) infiltration, and the formation of synovial tertiary lymphoid structures (TLS), whereas such adaptive immune markers are notably absent in OA and SNRA (58). In SPRA synovium, Tfh/Tph-B cell clonal expansion is prominent (59), while B cell activation and differentiation in OA are more complex (9); SPRA is driven by IL-21 and BAFF-mediated B cell activation (20), whereas OA is primarily mediated by IL-1β and IL-6-driven innate immunity (60). Consequently, SPRA can benefit from B cell depletion therapies (e.g., rituximab) or JAK inhibitors, whereas OA involves a balance of Th1/Th2/Th17/Treg cells, M1/M2 macrophages, and B cell activation/regulatory B cells (Breg). Targeting OA is more complicated, as the elimination of a specific cell subset may disrupt other immune cells and even exacerbate OA progression. Therefore, directly applying RA-targeted immunotherapies to OA lacks sufficient theoretical support, and future research requires a deeper exploration and discussion of the complex immune microenvironment in OA.

In summary, although both osteoarthritis and rheumatoid arthritis are associated with the immune system, their immunological mechanisms and treatment approaches differ significantly. Whether in basic research or clinical practice, it is essential to distinguish the type of arthritis (OA, SNRA, or SPRA) to propose more rational research or treatment strategies.

## 5 Existing targeted immune therapy strategies in osteoarthritis

Immunotherapy for osteoarthritis is a targeted therapeutic approach aimed at alleviating inflammation and joint pain by modulating immune responses or targeting specific cellular signaling pathways. Below are some immunotherapeutic strategies for osteoarthritis:

### 5.1 Biologics and targeted drug therapies

Currently, the biologics for targeted treatment of osteoarthritis mainly focus on targeting key inflammatory factors. The relevant targeted drugs are capable of specifically neutralizing certain cytokines without causing additional significant side effects, making them promising drugs for the treatment of persistent inflammation (61), They also hold great potential for application in osteoarthritis (OA). Numerous studies in experimental animal models have focused on targeting cytokines such as tumor necrosis factor (TNF) and interleukin-6 (IL-6) for OA treatment, including drugs like Etanercept, Infliximab, and Adalimumab (62). However, in clinical trials, TNF inhibitors (e.g., Infliximab) have shown less efficacy in OA compared to rheumatoid arthritis (RA), possibly due to the lower levels of inflammation and heterogeneity in OA. Additionally, the IL-6 receptor inhibitor Tocilizumab has entered clinical trials, but early results indicate limited improvement in pain and function for OA patients, with no significant delay in radiographic progression (63).

In light of the mixed results from clinical trials of TNF and IL-6 inhibitors in OA, some researchers suggest that their limited efficacy may be related to their weaker mechanistic role in OA. These drugs might only be effective for specific subtypes of OA, such as inflammatory OA with evident synovitis or elevated systemic inflammatory markers. Broader application in OA requires more precise patient stratification and longer-term clinical trial validation. Based on our summary and analysis of OA’s immune mechanisms, many other factors may influence the clinical trial outcomes of biological agents, including the complex etiology of patients, disease duration, the unique immune microenvironment within OA joints, and the critical roles and balance of T cells and B cells in OA. Additionally, the use of intravenous administration for these biological agents limits their concentration and aggregation within the joint, potentially affecting clinical efficacy. Future clinical trials could explore alternative methods, such as intra-articular injections. Further research should focus on conducting more clinical trials targeting specific OA subtypes (e.g., inflammatory OA) and investigating the combined use of TNF and IL-6 inhibitors with other therapies, such as stem cell therapy and chondroprotective agents.

### 5.2 Stem cell therapy

Stem cell therapy is a cutting-edge immunotherapeutic approach that involves the injection of stem cells to repair damaged knee joint tissues. Stem cells possess multipotent differentiation potential and can differentiate into joint tissue cells, such as chondrocytes, thereby repairing damaged articular cartilage and surrounding tissues (64). For example, mesenchymal stem cells (MSCs) are a promising treatment for mild to moderate knee osteoarthritis (65–70), Intra-articular MSC injections can reduce intra-articular inflammatory cells, promote cartilage regeneration, and further alleviate pain and improve joint function in patients (71–74). Currently, ongoing clinical trials include NCT02580695, NCT03166865, and NCT03818737. Researchers believe that MSC therapy holds significant potential for OA treatment, but further optimization of preparation methods, validation of long-term efficacy, and cost reduction are needed. Future research directions should focus on optimizing MSC preparation, exploring combination therapies, developing novel MSC-based approaches, and identifying biomarkers to screen patients who may benefit from MSC therapy, thereby enabling personalized treatment.

### 5.3 Comprehensive immunotherapy strategies

In addition to the specific immunotherapeutic approaches mentioned above, comprehensive immunotherapy strategies can be adopted to optimize treatment outcomes. These include:

- Personalized Treatment Plans: Tailoring individualized treatment plans based on the patient’s specific conditions, including selecting appropriate immunotherapeutic drugs, dosages, and treatment durations.
- Combination Therapies: Integrating immunotherapeutic drugs with other treatment modalities, such as physical therapy, rehabilitation exercises, and pharmacological treatments, to form a comprehensive treatment strategy and enhance therapeutic efficacy.

In summary, immunotherapeutic strategies for knee osteoarthritis include biological agents, targeted drug therapies, stem cell therapy, and comprehensive immunotherapy approaches. Each method has its unique characteristics, and patients should choose the most suitable treatment plan under the guidance of professional physicians. Additionally, maintaining healthy lifestyle habits and exercise routines is crucial for the prevention and management of knee osteoarthritis.

Although OA and RA differ in their pathogenesis, clinical diagnosis, and damage characteristics, both conditions manifest as joint damage and inflammation. Therefore, despite the current instability in the clinical trial outcomes of immunotherapies for OA, the prospect of modulating immune responses to delay OA progression remains promising. For RA, the pathogenesis driven by immune dysregulation is relatively clear, leading to well-established clinical treatment protocols. However, simply replicating RA treatment experiences and strategies for OA is unscientific, as OA has diverse pathogenic mechanisms, and the inflammation and immune dysfunction caused by different triggers vary significantly. This complexity involves the roles of various immune cells, such as macrophages, neutrophils, T cells, and B cells. Although no mature immunotherapeutic regimen for OA has been established, the indiscriminate elimination of a specific immune cell subset may lead to unexpected OA damage and other immune-related side effects, such as infections, which is one of the key reasons for the suboptimal outcomes in current clinical trials. However, with advancements in omics and single-cell technologies, scientists are gaining a deeper understanding of the immune landscape in OA. Correspondingly, the roles of various immune cell populations in OA are becoming clearer, and the therapeutic potential of targeting specific immune cell subsets is increasingly evident. This aligns with the current clinical concept of precision medicine and offers the promise of new clinical solutions with both efficacy and safety for OA treatment. Future research should further explore the dynamic changes in immune cell subsets, the interaction mechanisms between immune cells and chondrocytes, and the feasibility of combination therapy strategies.

## 6 M1 macrophages promote cartilage damage, whereas M2 macrophages may serve as potential targets for cartilage regeneration

**Figure 1:**
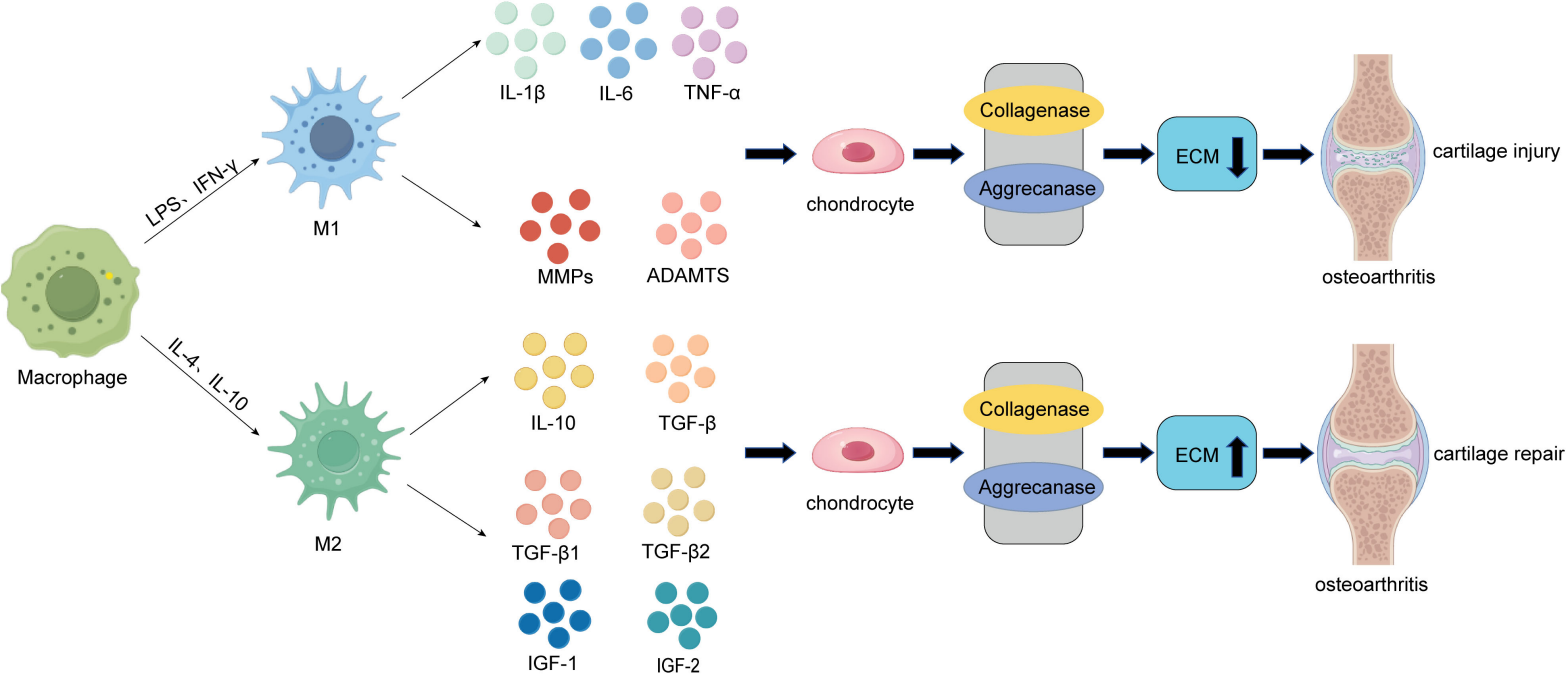
The role of macrophages in the pathogenesis of cartilage damage and repair in OA. On one hand, during the early inflammatory phase of osteoarthritis, macrophages within the joint can be polarized into pro-inflammatory M1 macrophages under the influence of LPS and IFN-γ. These M1 macrophages secrete pro-inflammatory cytokines such as IL-1β, IL-6, and TNF-α, as well as cartilage-degrading factors like MMPs (MMP-1, MMP-13) and ADAMTs. These factors act on chondrocytes, inducing the degradation of the extracellular matrix (ECM) through the activation of collagenases and aggrecanases, ultimately leading to cartilage damage and the progression of osteoarthritis (OA). On the other hand, during the late-stage cartilage destruction phase of OA, macrophages within the joint can be polarized into anti-inflammatory M2 macrophages under the influence of IL-4 and IL-10. These M2 macrophages secrete anti-inflammatory cytokines such as IL-10 and TGF-β, as well as cartilage-promoting factors like TGF-β1, TGF-β3, IGF-1, and IGF-2. By inhibiting the activity of collagenases and aggrecanases, M2 macrophages enhance ECM secretion by chondrocytes, ultimately promoting compensatory cartilage repair.

Macrophages are the most abundant immune cells in the knee joint and are present in the synovial lining along with fibroblasts (7). The primary role of macrophages is to phagocytose, kill, and eliminate pathogens, maintaining the body’s cleanliness and normal function. Additionally, macrophages play a significant role in immune regulation by secreting cytokines to modulate the functions of other immune cells or parenchymal cells. When articular cartilage is damaged, the infiltration of monocytes and macrophages increases in the synovial tissue and joint fluid (7, 75, 76). Macrophages initially phagocytose cartilage debris and secrete inflammatory mediators to stimulate the recruitment of other immune cells, thereby accelerating the clearance of damaged tissue (10). However, as inflammation worsens, macrophages can polarize from a resting state into M1 and M2 phenotypes, exerting distinct functions (77) (**Figure 1**).

During cartilage injury, macrophages play a crucial role. In the early stages of osteoarthritis (OA), M0 macrophages are polarized into M1 macrophages under the influence of interferon-gamma (IFN-γ), lipopolysaccharide (LPS), and tumor necrosis factor-alpha (TNF-α), promoting inflammation and exacerbating cartilage damage. Fahy et al. (78) noted that M1-associated cytokines such as interleukin-6 (IL-6), interleukin-1β (IL-1β), and TNF-α downregulate the synthesis of type II collagen and aggrecan, leading to cartilage degradation (**Figure 1**). Synovial M1 macrophages also promote the synthesis and secretion of proteolytic enzymes, such as matrix metalloproteinases (MMPs) including MMP-1, MMP-3, MMP-13, MMP-9, ADAMTS (a disintegrin and metalloproteinase with thrombospondin motifs), and cyclooxygenase-2 (COX-2). These enzymes are critical components driving cartilage degradation and can further aggravate cartilage damage (78–80) (**Figure 1**). Additionally, studies have shown that synovial macrophages and monocyte-derived pro-inflammatory macrophages negatively impact the chondrogenic potential of mesenchymal stem cells (MSCs) (81). Therefore, M1 macrophages, which express CD80, CD86, CD40, and MHC-II, contribute to inflammation, cartilage damage, and OA progression. M1 macrophage inhibitors may serve as novel immunotherapeutic targets.

In contrast to M1 macrophages, which primarily act during the early stages of osteoarthritis (OA), M2 macrophages exhibit anti-inflammatory properties and play a crucial role in promoting cartilage repair and regeneration (7, 16). Studies have shown that M2 macrophages can secrete interleukin-10 (IL-10), transforming growth factor-beta (TGF-β), interleukin-1 receptor antagonist (IL-1RA), chemokine (C-C motif) ligand 18 (CCL18), and other anti-inflammatory mediators. Additionally, they release cartilage-promoting cytokines such as TGF-β1, TGF-β3, insulin-like growth factor 1 (IGF-1), and IGF-2, tilting the local immune microenvironment toward a pro-chondrogenic state, thereby facilitating cartilage repair and regeneration and delaying OA progression (7, 82) (**Figure 1**). Furthermore, M2 macrophages have been reported to secrete vascular endothelial growth factor (VEGF), TGF-β, and arginine, promoting the synthesis of collagen and proteoglycans, which enhances cartilage regeneration (83, 84). Research by Dai et al. demonstrated that certain biomaterials can induce M2 macrophage polarization, leading to the release of regulatory cytokines and exerting immunomodulatory effects on tissue healing (82). Additionally, studies by Kai Zhou et al. revealed that M2H@RPK can provide inflammation-targeted therapy through macrophage repolarization, alleviating synovitis and cartilage damage caused by OA (85).

In summary, macrophages are critical for promoting OA progression in the early stages, but their polarization toward the M2 phenotype in later stages can help mitigate OA or repair damaged tissues. Therefore, completely eliminating macrophages throughout the course of OA is not scientifically sound. However, strategies such as early-stage macrophage depletion or functional inhibition, or timely induction of M1-to-M2 polarization during OA, could be promising therapeutic approaches. Nonetheless, precise timing and dosage of macrophage intervention are essential for achieving optimal outcomes.

## 7 IL-1β and elastase released by neutrophils exacerbate cartilage damage, whereas their derived extracellular vesicles enhance cartilage protection

Neutrophils are the first immune cells recruited during cartilage damage in osteoarthritis (OA). They secrete pro-inflammatory mediators and elastase, which induce chondrocyte apoptosis and extracellular matrix (ECM) degradation, thereby promoting the progression of OA (8, 10, 86).

**Figure 2:**
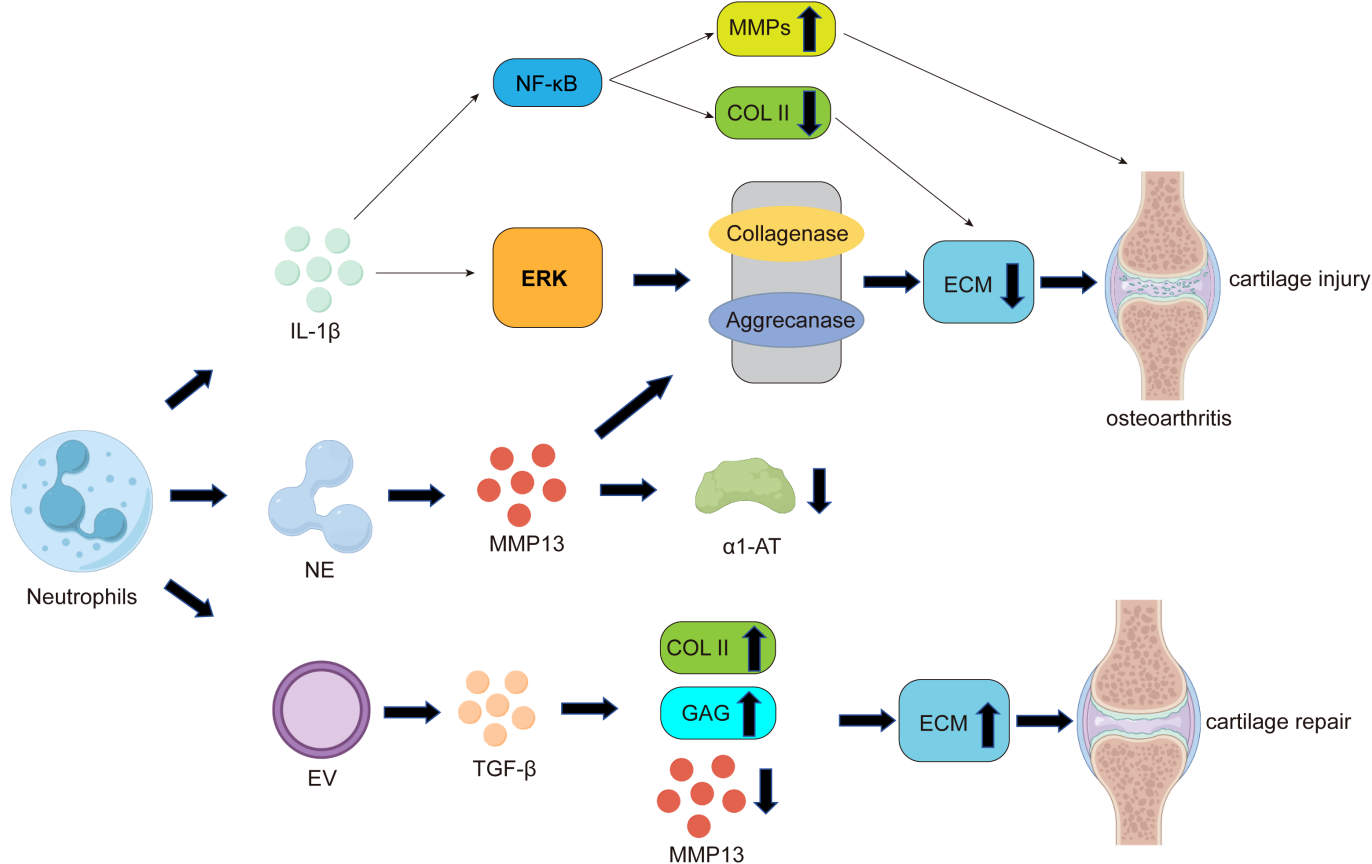
The role of neutrophils in the pathogenesis of cartilage damage and repair in OA. Similar to macrophages, neutrophils can secrete pro-inflammatory factors such as IL-1β. On one hand, IL-1β can activate nuclear factor-kappa B (NF-κB), promoting the expression of matrix metalloproteinases (MMPs) while suppressing the expression of type II collagen (COL2), leading to reduced extracellular matrix (ECM) secretion by chondrocytes. On the other hand, IL-1β can activate extracellular signal-regulated kinase (ERK) to decrease cartilage ECM production, ultimately resulting in cartilage damage and the development of osteoarthritis. Additionally, one of the most significant ways neutrophils contribute to cartilage damage is through the release of neutrophil elastase (NE). NE can activate MMP-13, the most critical collagenase responsible for cartilage degradation during OA. As mentioned earlier, MMP-13 can induce ECM degradation by activating collagenases and aggrecanases. Furthermore, activated MMP-13 can inhibit the proteolytic activity of its own inhibitor, alpha-1 antitrypsin (α1-AT), ultimately leading to cartilage damage. Notably, neutrophil-derived extracellular vesicles (EVs) can promote increased ECM secretion by chondrocytes through the upregulation of COL2 expression and the induction of transforming growth factor-beta (TGF-β) production, ultimately facilitating cartilage repair.

On one hand, similar to macrophages and other immune cells, neutrophils can secrete interleukin-1β (IL-1β), which exacerbates cartilage damage in OA through multiple pathways. For example, IL-1β can activate extracellular signal-regulated kinase (ERK) to reduce the production of cartilage ECM (87, 88). Additionally, IL-1β can downregulate the expression of antioxidant enzymes that scavenge reactive oxygen species (ROS), including superoxide dismutase, catalase, and glutathione peroxidase, thereby accelerating ROS-mediated destruction of articular cartilage (89). IL-1β induces ECM degradation by activating collagenases and aggrecanases (90) (**Figure 2**), leading to chondrocyte hypertrophy and dedifferentiation, and ultimately resulting in chondrocyte apoptosis. IL-1β can also promote OA progression by activating nuclear factor-kappa B (NF-κB) (91), When NF-κB is activated, it regulates chondrocyte hypertrophy through cytokines such as SRY-box transcription factor 9 (SOX9), bone morphogenetic protein 2 (BMP2), matrix metalloproteinases (MMPs), and HIF-2α (92), while suppressing the expression of type II collagen (COL2) and disrupting chondrocyte metabolism (**Figure 2**).

On the other hand, one of the most significant ways neutrophils contribute to cartilage damage is through the release of tissue-destructive proteases, among which neutrophil elastase (NE) is considered the primary protease involved in cartilage damage and inflammatory destruction in osteoarthritis (OA) (93). NE can activate matrix metalloproteinase-13 (MMP-13), the most critical collagenase responsible for cartilage degradation during OA (94). Elastase-activated MMP-13 leads to the proteolysis of its own inhibitor, alpha-1 antitrypsin (α1-AT), and abnormal cartilage degradation (**Figure 2**). Studies have shown that α1-AT promotes the transcription of COL2A1, ACAN, and SOX9 genes while downregulating the expression of MMP13 and ADAMTS5 genes, providing a protective effect against joint inflammation and cartilage degradation (95). Additionally, research has found that elastase disrupts the cartilage matrix and induces the release of peptidylarginine deiminase 2 (PADI2) from fibroblast-like synoviocytes (FLS) (96).

Notably, a few studies suggest that neutrophils can act as immune response modulators in immune-related diseases, offering some protective effects against cartilage damage. Neutrophil-derived extracellular vesicles (EVs) can exert anti-inflammatory effects (97) and provide cartilage protection by increasing type II collagen and reducing the expression of type X collagen within the joint. Therefore, neutrophil EVs could be developed as an autologous therapy to protect and repair joint tissues in patients affected by inflammatory arthritis (98). Further exploration has confirmed that neutrophil vesicles enhance cartilage protection by inducing the production of transforming growth factor-beta (TGF-β), a key mediator of chondrocyte homeostasis. This stimulates the deposition of type II collagen (COL2) and glycosaminoglycans (GAGs) while downregulating cartilage-degrading enzymes(MMPs) (99, 100)(**Figure 2**). In summary, these studies indicate that neutrophils, on one hand, can accelerate cartilage damage and OA progression by secreting elastase(NE) and IL-1β. On the other hand, their derived extracellular vesicles can act as immune response modulators to protect against cartilage damage and promote cartilage repair (**Figure 2**).

Therefore, when targeting neutrophils for therapeutic intervention, it is essential to supplement the extracellular matrix in a timely manner to prevent potential cartilage loss. More importantly, interventions targeting neutrophils should be localized, as neutrophils play a critical role in systemic immune defense. Complete or systemic depletion of neutrophils may increase the risk of infections and other complications in patients.

## 8 Mature dendritic cells tend to induce inflammatory responses that promote cartilage degradation, while immature DCs can enhance immunoregulatory responses to protect cartilage

Dendritic cells (DCs) are a cluster of antigen-presenting cells that regulate inflammatory responses by secreting cytokines and inflammatory mediators associated with chronic inflammation (101). Studies have shown that DCs are primarily found in synovial fluid, mainly as mature cDCs (conventional DCs derived from myeloid stem cells) and immature pDCs (plasmacytoid DCs derived from lymphoid stem cells), and play a unique role in cartilage damage (102). Additionally, a significant number of DCs have been observed in the synovium during the early stages of rabbit OA models. In the same early phase, the number of DCs increases markedly with the progression of synovial inflammation grading. The expression of IL-1β and TNF-α is also elevated in the early stages and subsequently decreases as synovial inflammation subsides (102).

Mature DCs contain activated inflammatory TLRs and release high levels of inflammatory mediators, inducing the proliferation of Th1 and Th17 cells and targeting self-antigens, thereby directly or indirectly inhibiting chondrogenesis and promoting cartilage degeneration through the action of inflammatory MSCs (103, 104). In contrast, immature DC populations tend to enhance regulatory responses. They can modulate immune regulation in inflamed joints by releasing IL-10, which promotes the proliferation of regulatory T cells (Tregs) and stimulates the chondrogenic differentiation of MSCs (105). Therefore, mature DCs are prone to secreting inflammatory factors that directly or indirectly exacerbate cartilage damage, while immature DCs tend to release IL-10 to enhance regulatory responses and strengthen cartilage protection.

Do dendritic cells (DCs) also hold potential therapeutic value in the treatment of arthritis? Research indicates that regulatory dendritic cells (DC-regs) have been widely used in the treatment of autoimmune diseases and arthritis (NCT04303208, NCT04303208, NCT03337165), and DC-regs have shown promise as a therapeutic tool for rheumatoid arthritis (RA) and other immune-inflammatory diseases at the animal model level (106, 107). Therefore, some scholars have proposed the use of induced regulatory autologous plasmacytoid DCs (pDCs) via intra-articular injection for the treatment of OA (104). While these cells possess the potential to modulate immune responses, their specific therapeutic efficacy still requires further investigation.

## 9 T cells regulate chondrocyte function and homeostasis by secreting cytokines and growth factors

In the OA microenvironment, T cells regulate chondrocyte function and homeostasis by secreting cytokines and growth factors (108). Following cartilage injury, local immune responses are activated, and T cells are recruited to the injury site, releasing cytokines to modulate the immune response. Different T cell subtypes play distinct roles in cartilage damage. Studies have shown that the infiltration of CD3^+^ T, CD4^+^ T, and CD8^+^ T cells is significantly increased in the synovial fluid and synovial tissue of patients with cartilage damage (41, 109, 110).

**Figure 3:**
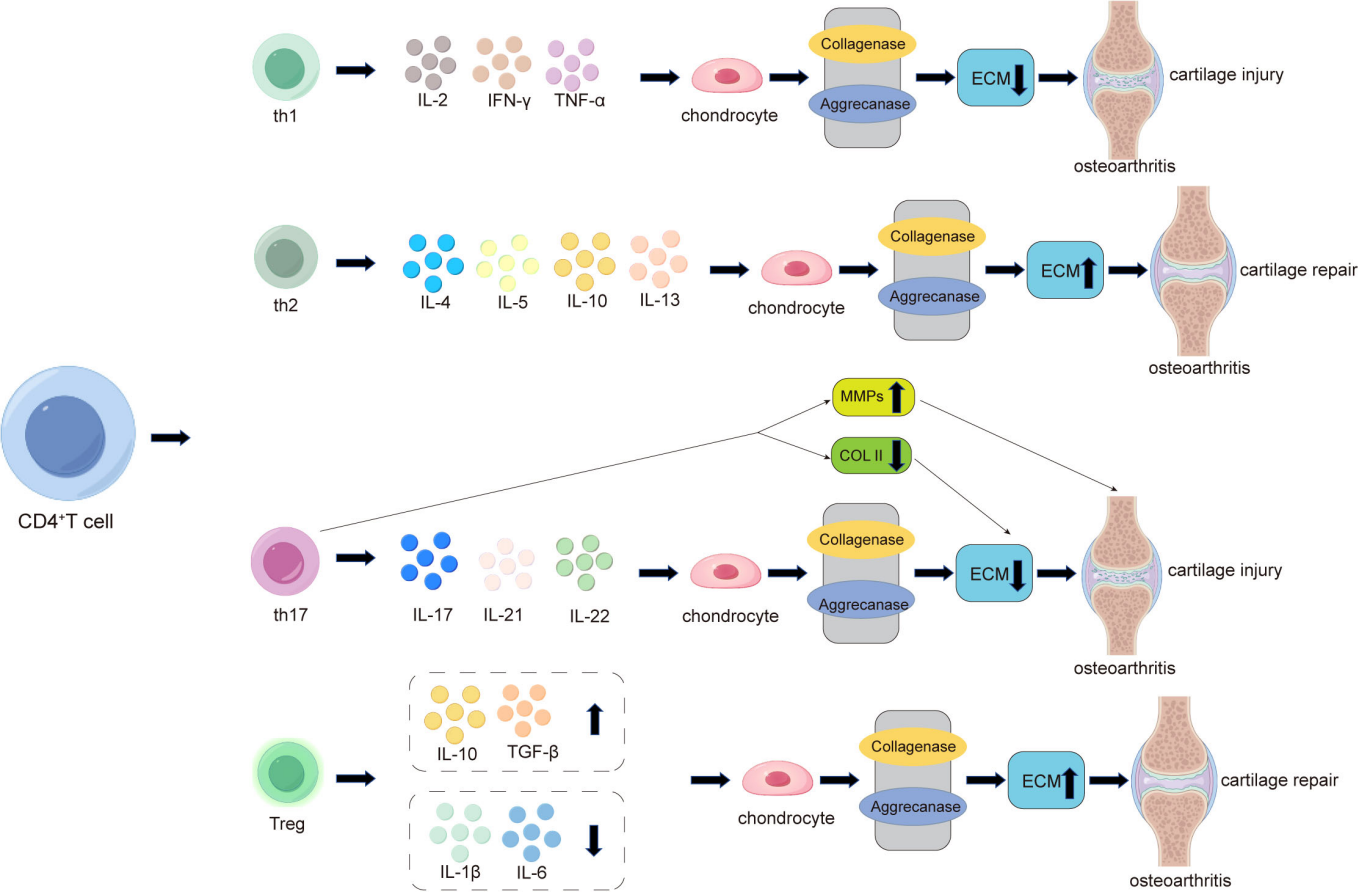
The role of CD4^+^ T cells in the pathogenesis of cartilage damage and repair in osteoarthritis (OA). In OA, activated CD4^+^ T cells differentiate into four subtypes: Th1, Th2, Th17, and Treg. Th1 cells primarily promote local inflammation and cartilage damage in OA by secreting pro-inflammatory factors such as IL-2, IFN-γ, and TNF-α, while Th2 cells alleviate local inflammation and promote cartilage repair by secreting anti-inflammatory factors such as IL-4, IL-5, IL-10, and IL-13. Additionally, Th17 cells play a significant role in OA by secreting pro-inflammatory factors such as IL-17, IL-21, and IL-22. Among these, the most critical inflammatory cytokine, IL-17, acts on chondrocytes to inhibit proteoglycan production, thereby suppressing cartilage repair and exacerbating cartilage damage. Notably, Th17 cells also enhance the expression of cartilage-degrading factors such as MMP-1, MMP-3, and MMP-13 in chondrocytes while reducing the expression of type II collagen (COL2), leading to decreased extracellular matrix (ECM) secretion by chondrocytes and ultimately worsening cartilage damage. In the late stages of arthritis, CD4^+^ T cells increasingly differentiate into regulatory T cells (Tregs). Activated Tregs promote the secretion of anti-inflammatory molecules, including IL-10 and TGF-β, while inhibiting the production of IL-1β and IL-6, thereby maintaining the homeostasis of the regenerative microenvironment and compensatorily mitigating cartilage damage.

Antigen-activated CD4^+^ T cells are primarily divided into four subtypes: T helper 1 (Th1), T helper 2 (Th2), T helper 17 (Th17), and regulatory T cells (Tregs). Th1 cells mainly secrete IL-2, IFN-γ, and TNF-α, participating in the regulation of cellular immunity and macrophage activation (**Figure 3**). IL-2 and TNF-α secreted by Th1 cells can activate osteoclasts (111). The pro-inflammatory effects of IFN-γ and TNF-α induce chondrocyte apoptosis and cartilage matrix breakdown, leading to the formation of osteophytes (18, 112). CD4^+^ T cells differentiate into Th2 cells under the influence of cytokines such as IL-4 (18). Th2 cells release cytokines such as IL-4, IL-5, IL-10, and IL-13, which are inherently anti-inflammatory (**Figure 3**). Therefore, Th1 and Th2 cell responses are considered pro-inflammatory and anti-inflammatory, respectively (113). One study demonstrated that calcitriol can influence the differentiation of T cell subsets by inhibiting the proliferation of immature CD4^+^ T cells into Th1 cells and promoting Th2 cell maturation, thereby affecting the balance between osteoblasts and osteoclasts (114). Additionally, macrophages and dendritic cells secrete cytokines such as IL-4, promoting Th2 differentiation through various pathways (115). Research has shown that an imbalance between Th1 and Th2 cells is associated with the pathogenesis of osteoarthritis, contributing to inflammation and disease progression (30). Furthermore, Th17 cells represent a unique and important subset of T cells. Their function depends on the immune system’s ability to produce and secrete key cytokines such as IL-17, IL-21, and IL-22 (116). Among these, the inflammatory cytokine IL-17 can act on chondrocytes and inhibit proteoglycan production, thereby suppressing cartilage repair (117). Notably, Th17 cells can also enhance the expression of MMP-1, MMP-3, and MMP-13 in chondrocytes while reducing the expression of tissue inhibitors of metalloproteinases (TIMP-2), type II collagen, proteoglycans, and link proteins, all of which promote cartilage degradation and exacerbate cartilage damage (118) (**Figure 3**). Regulatory T cells (Tregs) are a CD4^+^ T and CD25^+^ subset of T lymphocytes with anti-inflammatory properties (119). Studies have shown that activated Tregs promote the secretion of anti-inflammatory molecules, including IL-10, TGF-β, and indoleamine 2,3-dioxygenase (IDO), while inhibiting the production of IL-1β and IL-6, thereby maintaining the homeostasis of the regenerative microenvironment and indirectly promoting tissue regeneration (120) (**Figure 3**).

Research has found that abnormal immune dysregulation are a key factor in the progression of rheumatoid arthritis (RA) (46). Immune dysregulation partially depend on the assistance of T cells, specifically follicular helper T cells (Tfh), with those circulating in the peripheral blood referred to as peripheral helper T cells (Tph) (42, 121). Studies by Rao DA et al. have shown a significant increase in both Tph and Tfh cells in RA. Tph cells promote B cell activation and the formation of tertiary lymphoid structures by highly expressing CXCL13, IL-21, and PD-1 (122). However, to date, no studies have clearly elucidated the role of Tph in osteoarthritis (OA), and whether B cell-secreted antibodies can promote OA progression requires further evidence. Tfh cells can release cytokines such as IL-4 and IL-21, which may influence OA progression under certain conditions (59, 122, 123). Therefore, whether the same strategies targeting Tfh/Tph for RA treatment can be applied to OA needs careful consideration, as this is related to the functional heterogeneity of the same cell subsets in different diseases. Future research could utilize single-cell transcriptomic data from OA patient synovium to identify Tph-specific gene modules and validate the impact of Tph cell deficiency on cartilage degeneration using Tph gene knockout mouse models of OA.

Additionally, CD8^+^T cells play an important role in OA cartilage damage. Studies have shown that during OA, CD8^+^ T cells are activated and constitutively proliferate in the progression of OA in mouse ACLT models, exacerbating cartilage damage by expressing tissue inhibitor of metalloproteinases-1 (TIMP-1) (110).

Therefore, when investigating T cell-targeted therapies, it is not advisable to indiscriminately eliminate CD4^+^ T cells using antibodies, as the balance between Th1, Th17, and Treg cells is crucial for OA. The depletion of CD4^+^ T cells, which includes the elimination of Treg cells, can lead to osteoporosis, abnormal immune environments within the bone, and potentially trigger conditions such as enteritis or skin inflammation during treatment. In contrast, localized depletion of CD8^+^ T cells has been shown to alleviate OA (110). Recent research on CD8^+^ T cells in tissue inflammation has been increasing, including their role in fatty liver inflammation and their association with asthma, making targeting CD8^+^ T cells highly valuable (124, 125). While some scientists may be concerned about the additional risks associated with eliminating CD8^+^ T cells, such as infections, the antibody responses generated by B cells can still maintain the body’s normal immune reactions.

## 10 B cells regulate extracellular matrix degradation by secreting cytokines and modulating immune responses

B cells are essential components of humoral immunity, functioning in antigen presentation and the secretion of antibodies. Studies have shown that B cells also play a role in the progression of osteoarthritis (OA), as their proliferation and differentiation capabilities are altered in the synovium of OA patients (9). In terms of cartilage damage, B cells regulate ECM degradation by secreting cytokines and modulating immune responses. On one hand, B cells promote cartilage degradation by secreting pro-inflammatory substances and antibodies, while on the other hand, they aid in cartilage repair by controlling autoimmune responses.

**Figure 4:**
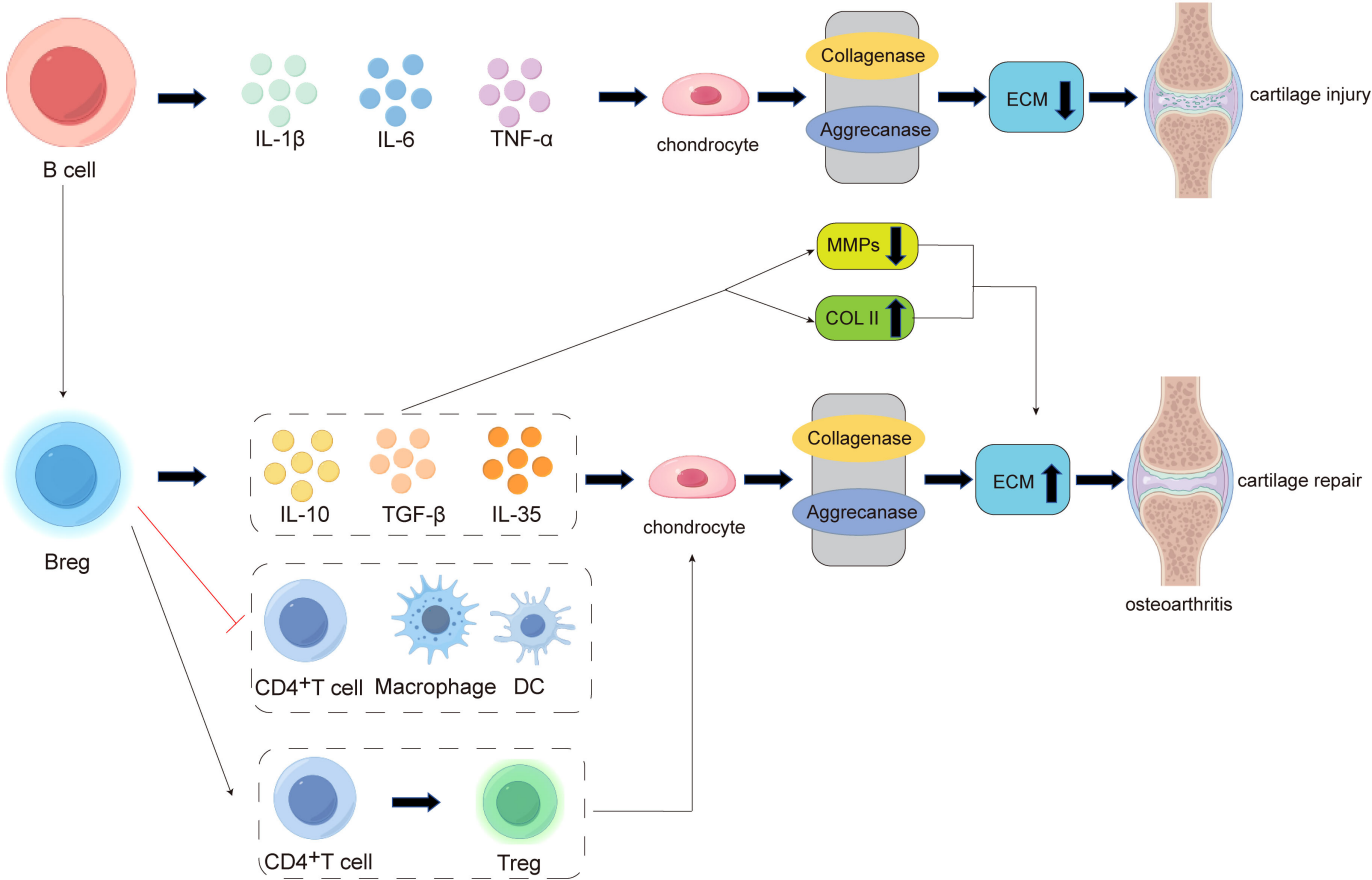
The pathogenesis of B cell involvement in cartilage damage and repair in osteoarthritis (OA). In the early stages of inflammation, B cells can secrete pro-inflammatory factors such as IL-1β, IL-6, and TNF-α, which act on chondrocytes to enhance the activity of collagenases and aggrecanases. This leads to a reduction in extracellular matrix (ECM) secretion and promotes cartilage damage. In the late stage of cartilage destruction, there is an increased differentiation of B cells into regulatory B cells (Bregs). On one hand, Bregs can secrete anti-inflammatory factors such as IL-10, TGF-β, and IL-35, promoting chondrocytes to produce more ECM. On the other hand, Bregs can suppress the expansion of pathogenic T cells (e.g., CD4^+^ T cells) and other pro-inflammatory lymphocytes (e.g., macrophages and dendritic cells), while also inducing the differentiation of CD4^+^ T cells into regulatory T cells (Tregs). These mechanisms collectively exert immunomodulatory effects, ultimately promoting cartilage repair.

Research indicates that B cells can secrete various pro-inflammatory factors, including interleukin-1β (IL-1β), interleukin-6 (IL-6), and tumor necrosis factor-alpha (TNF-α), which induce chondrocyte death and cartilage matrix destruction (126) (**Figure 4**). IL-1β, IL-6, and TNF-α can inhibit the synthesis of type II collagen (Col II) and glycosaminoglycans (GAGs,such as Chondroitin sulfate, Keratan sulfate, and Hyaluronic acid) while upregulating the production of cartilage-degrading enzymes such as matrix metalloproteinases (MMP-1, MMP-3, and MMP-13), thereby promoting ECM degradation (127) (**Figure 4**).

Recent studies have found that within the joint, there exists a unique B cell subpopulation with special functions. These cells are involved in the regulation of immune responses, mediating immune tolerance, and exerting immunosuppressive effects. This protective B cell subpopulation is known as regulatory B cells (Bregs) (17). There are two main pathways through which Bregs inhibit inflammation: First, by producing anti-inflammatory cytokines (such as IL-10, IL-35, and TGF-β) to negatively regulate immune responses (17) (**Figure 4**). IL-10 is an anti-inflammatory cytokine produced by immune cells such as T cells, macrophages, and B cells. It inhibits the secretion of pro-inflammatory cytokines by T cells and the antigen-presenting functions of dendritic cells (DCs) and macrophages. IL-35 Breg cells can negatively regulate the antigen-presenting functions of macrophages, inflammatory T cells, and B cells (128), while also expanding Treg cells (129). TGF-β can induce the differentiation of naïve CD4^+^ T cells into Treg cells and transform immature DCs into tolerogenic DCs. Additionally, TGF-β Breg cells express CD5 *in vitro* and induce the differentiation of naïve CD4^+^ T cells into functionally suppressive Treg cells (130). Second, by inhibiting the proliferation of pathogenic T cells (such as CD4^+^ T cells, cytotoxic CTLs, etc.) and other pro-inflammatory lymphocytes (such as macrophages, dendritic cells, etc.), while promoting the differentiation of regulatory T cells (Tregs) (17), thereby reducing cartilage damage (**Figure 4**).

In summary, B cells and their subset, Bregs, can modulate the degradation and formation of the extracellular matrix (ECM) through the secretion of cytokines and regulation of immune responses. Therefore, when studying B cell-targeted therapies for osteoarthritis (OA), the complexity arises from the balance between B cell activation and Bregs within the joint. Directly targeting and depleting total B cells may disrupt other B cell subsets or other immune cell populations, potentially exacerbating OA progression. Investigating the conditions under which B cells can be promoted to differentiate into Bregs, thereby enhancing Bregs’ ability to secrete cytokines like IL-10 to promote ECM formation and cartilage repair, is a scientific question that requires further exploration. This could provide new strategies for the clinical treatment of cartilage damage in osteoarthritis.

## 11 Activated mast cells induce cartilage degradation and exacerbate OA cartilage damage

Mast cells are round or oval granulocytes present in tissues and organs throughout the body. As sentinels of the innate immune system, mast cells are poised to respond rapidly to exogenous pathogens and endogenous danger signals. Recent studies have shown that mast cells influenced by the synovial microenvironment exhibit an activated phenotype, which can impact the progression of rheumatoid arthritis and exacerbate cartilage damage (131). In osteoarthritis (OA), research has also demonstrated that IgE-mediated mast cells can be activated via FcϵRI and Syk, leading to mast cell degranulation and the release of pro-inflammatory factors and cartilage-degrading mediators (including tryptase), resulting in cartilage and joint destruction and aggravating OA cartilage damage (13). Taylor et al. found that activated mast cells release histamine, inducing chondrocytes to produce cartilage-degrading factors such as MMP-13 and prostaglandin E2 (PGE2), leading to the degradation of proteoglycans and type II collagen, and ultimately causing extracellular matrix (ECM) degradation (132). Similarly, studies have shown that mast cell-derived tryptase can degrade cartilage ECM components and activate MMP13, thereby inducing joint inflammation, chondrocyte apoptosis, and cartilage destruction (13, 133). Additionally, another study found that co-culturing activated mast cells with chondrocytes resulted in proteoglycan degradation and subsequent joint cartilage damage (134).

Recent studies have revealed that degranulated mast cells mediate inflammatory signaling through neutrophil trapping and endocytosis, triggering acute inflammatory responses (135). Furthermore, mast cells regulate T cell responses in arthritis animal models by promoting the proliferation of CD4^+^ T cells and Th1/Th17 cytokines (136). These studies suggest that mast cells can recruit other immune cells, leading to cartilage degradation and subchondral bone remodeling.

Therefore, mast cells play a significant role in cartilage degradation. Inhibiting mast cell activation through drugs or inhibitors targeting key genes may serve as a novel strategy for preventing and treating OA cartilage damage. Future research could further explore whether specific subsets of mast cells may alleviate OA, providing a more comprehensive understanding of the role of mast cells in OA progression and enabling the development of more holistic therapeutic strategies.

## 12 Dynamic changes and interactions of immune cells in the joint region of OA

### 12.1 Dynamic changes among immune cells in the OA joint

During the progression of osteoarthritis (OA), the dynamic changes in immune cells are a critical feature of disease development, spanning from the early inflammatory phase to the late cartilage destruction phase.

In the early inflammatory phase, macrophage infiltration and activation are predominantly characterized by M1-type macrophages. These macrophages secrete pro-inflammatory cytokines such as IL-1β and TNF-α, which not only promote intra-articular inflammation but also directly contribute to the degradation of the cartilage matrix (85, 137, 138). In addition to macrophages, the early stages of OA are accompanied by an increase in granulocytes and enhanced antigen-presenting capacity of dendritic cells (DCs). The rise in granulocytes leads to the release of cytokines such as IFN-γ, TNF-α, and IL-1β, recruiting other immune cells to infiltrate the joint. Recent studies have shown that neutrophils within tumors can also exhibit antigen-presenting functions, suggesting the need to explore whether granulocytes possess unknown roles in OA (139). The activation of innate immunity, cytokine production, and enhanced antigen presentation collectively facilitate the activation and differentiation of T cells. Studies have demonstrated significant expansion of Th1 and Th17 cells in OA, which exacerbate inflammation and cartilage damage through the secretion of IFN-γ and IL-17 (140). B cells may participate in the inflammatory response during the early stages by secreting antibodies and cytokines, although their specific mechanisms require further investigation.

In the late cartilage destruction phase, macrophages gradually polarize toward the M2 phenotype, secreting anti-inflammatory factors such as IL-10 to compensatorily alleviate inflammation (138, 141). However, by this stage, inflammation has already caused severe cartilage damage, and the anti-inflammatory effects of M2 macrophages may be insufficient to reverse the pathological progression. The proportion of regulatory T cells (Tregs) increases in the late stage, suppressing inflammation through the secretion of IL-10 and TGF-β (18, 142). Additionally, CD8^+^ T cells may enter an exhausted state, characterized by dysfunction and apoptosis. In addition to their classical functions in OA, regulatory B cells may also play a significant role within the joint, although the specific mechanisms involved require further investigation. Meanwhile, in advanced OA, neutrophils continue to be present at a high proportion, indicating that neutrophils can persistently exert their effects in OA (86).

### 12.2 Interactions among immune cells in the OA joint

Within the OA joint, immune cells interact through various mechanisms to collectively regulate inflammatory responses and cartilage destruction. For example, the interaction between macrophages and T cells primarily involves antigen presentation, cytokine release, and co-stimulatory molecule-mediated signaling to modulate inflammation and immune responses. As antigen-presenting cells (APCs), macrophages present antigens to T cells via MHC-II molecules while releasing IL-12 and IL-23 to promote the differentiation of Th1 and Th17 cells, exacerbating inflammation and cartilage damage (138, 143); In contrast, M2-type macrophages secrete IL-10 and TGF-β to promote Treg cell differentiation, suppress inflammation, and facilitate cartilage repair (138). Additionally, macrophages enhance T cell activation through the binding of co-stimulatory molecules (such as CD80/CD86) to CD28 on T cells (144). These dynamic interactions play a critical role in the progression of inflammation and tissue destruction in OA, while also providing potential directions for therapeutic strategies targeting immune cell interactions.

The interaction between macrophages and B cells primarily regulates B cell activation and function through cytokine release and direct cell contact. Macrophages secrete B cell-activating factors (BAFF) and IL-6 to promote B cell proliferation, differentiation, and antibody production, thereby exacerbating local inflammatory responses (145, 146). Furthermore, macrophages clear immune complexes produced by B cells via Fc receptors, preventing their deposition in the joint and reducing tissue damage (145). However, when antibodies produced by B cells bind to the surface of normal cells, they can induce macrophage attacks on healthy tissues, although this phenomenon is more common in rheumatoid arthritis (RA). In some cases, macrophages may further activate B cells through direct contact, such as CD40-CD40L interactions (146). These interactions play a significant role in the inflammatory microenvironment of OA, driving the persistence of inflammation and potentially regulating autoimmune responses, thus offering potential mechanisms for targeting macrophage-B cell interactions in OA treatment.

Within the OA joint, the interaction between T cells and B cells primarily regulates B cell activation and function through direct cell contact and cytokine-mediated signaling. T cells, particularly Th2 cells, provide co-stimulatory signals by binding CD40L on their surface to CD40 on B cells, promoting B cell differentiation into plasma cells and antibody production (147). Additionally, cytokines released by Th2 cells (e.g., IL-4, IL-5, IL-13) further regulate B cell proliferation, class switching, and antibody generation (146). In the inflammatory microenvironment of OA, this interaction may exacerbate local inflammation and contribute to the production of autoantibodies, thereby promoting joint destruction. Meanwhile, Treg cells attempt to maintain immune balance by secreting IL-10 and TGF-β to suppress excessive B cell activation. This dynamic T cell-B cell interaction plays a crucial role in the pathological progression of OA and provides a potential mechanism for therapeutic strategies targeting immune cell interactions.

## 13 Immune-mediated novel biomarkers and immunomodulatory strategies related to OA progression

Immune-mediated OA involves a variety of immune cells, inflammatory factors, and signaling pathways. The related novel biomarkers and emerging immunomodulatory strategies are currently hot topics in research. In terms of biomarkers, researchers are focusing on developing more sensitive and specific inflammatory markers (e.g., IL-1β, IL-6, TNF-α) and immune cell phenotypic markers (e.g., M1/M2 macrophage ratio, Treg/Th17 balance) to identify inflammatory OA subtypes and predict disease progression (148–150).

Regarding immunomodulatory strategies, emerging approaches such as multi-target cytokine inhibitors (e.g., simultaneously targeting IL-1β and TNF-α), immune cell-targeted therapies (e.g., promoting M2 macrophage polarization or Treg cell expansion), and metabolic reprogramming(Such as neutralizing mitochondrial ROS, protecting chondrocytes from oxidative stress damage) show great potential. These research directions are expected to provide new avenues for the precise diagnosis and personalized treatment of OA, thereby improving patient outcomes. Below is a summary table (**Table 1**) of some novel biomarkers and immunomodulatory strategies:

**Table 1:** Novel biomarkers and immunomodulatory strategies.

Category	Biomarker	Mechanism of Action	Targeted Regulatory Strategy
Inflammatory factors	IL-1β	Pro-inflammatory cytokines,Promote synovial inflammation, Promote cartilage apoptosis and degradation	IL-1 inhibitors: such as Anakinra
	TNF-α		TNF-α inhibitors: such as Infliximab
	IL-6		IL-6 inhibitors: such as Tocilizumab
	IL-17	Secreted by Th17 cells, Promote inflammation and cartilage destruction	IL-17 inhibitors
Matrix-degrading enzymes	MMP-3, MMP-13	Matrix metalloproteinases, Involved in cartilage matrix degradation	MMP inhibitors:such as Marimastat, inhibit the activity of matrix metalloproteinases
	ADAMTS-5	Primarily involved in proteoglycan degradation	ADAMTS-5 inhibitors: such as GLPG1972
Immune cells	CD4^+^Tcell, M1 macrophages	Pro-inflammatory effects	Macrophage polarization: Promote the differentiation of M2-type macrophages
	Treg, M2 macrophages	Anti-inflammatory and immunomodulatory	Treg cell expansion: Enhance Treg activity to suppress inflammation
Metabolites	PGE2、ROS	Promote inflammation and oxidative stress	Metabolic reprogramming(For example, neutralizing mitochondrial ROS to protect chondrocytes from oxidative stress damage.)
Epigenetic markers	MiRNA	Such as miR-140 and miR-146a, involved in chondrocyte metabolism and inflammation regulation	miRNA therapy:such as miR-140 mimics or inhibitors
	DNA methylation	The methylation status of certain genes may reflect OA progression	DNA methylation regulators: such as 5-Azacytidine
Extracellular vesicles	EVs	Inhibit the activity of inflammatory factors	Serve as drug delivery carriers, loading anti-inflammatory factors (such as IL-10, TGF-β) or small-molecule drugs (e.g., NSAIDs).

The table above lists seven major categories of immune-mediated osteoarthritis-related novel biomarkers, including inflammatory factors, matrix-degrading enzymes, immune cells, metabolic products, epigenetic markers, and extracellular vesicles. It also outlines their respective mechanisms of action and potential targeted immunomodulatory strategies.

Future research could integrate multi-omics studies, such as combining genomic, transcriptomic, proteomic, and metabolomic data, to comprehensively elucidate the immune mechanisms underlying OA. Based on patients’ immune profiles and biomarkers, precise and personalized treatment strategies can be developed.

## 14 Result and future perspectives

Cartilage damage is the most critical feature of osteoarthritis, caused by various factors, including acute trauma, chronic inflammation, and metabolic issues(Such as glucose metabolism, lipid metabolism, and immune-related metabolic inflammation.). This review systematically summarizes the dual roles and mechanisms of immune cell differentiation in cartilage damage during osteoarthritis (OA). Research indicates that immune cells (such as macrophages, neutrophils, dendritic cells, T cells, B cells, and mast cells) exhibit high plasticity and functional diversity within the OA joint microenvironment. On one hand, pro-inflammatory immune cells (e.g., M1-type macrophages, Th17 cells) directly or indirectly contribute to cartilage degeneration and extracellular matrix degradation by releasing inflammatory factors (e.g., TNF-α, IL-1β, IL-6) and matrix-degrading enzymes (e.g., MMPs, ADAMTS). On the other hand, anti-inflammatory and reparative immune cells (e.g., M2-type macrophages, regulatory T cells, and regulatory B cells) may exert protective effects by suppressing inflammatory responses and promoting tissue repair. Additionally, the interactions among immune cells and their crosstalk with chondrocytes further complicate the pathological progression of OA.

Currently, the treatment of OA is largely limited to exploratory attempts based on therapeutic strategies derived from the understanding of autoimmune diseases or rheumatoid arthritis (RA). However, due to the complex pathogenesis of OA and its unique intra-articular immune landscape, these empirical approaches have not achieved sufficiently favorable clinical outcomes in OA. Existing immunotherapeutic drugs also have certain limitations, including immune-related toxic side effects. For example, TNF inhibitors (such as infliximab, adalimumab, and etanercept) may induce anti-drug antibodies (ADAs) or autoimmune diseases (e.g., lupus-like syndrome) during the treatment of OA, which restricts their use in OA management. In the future, a deeper understanding of the immune microenvironment in OA and the identification of key immune cell subsets, cytokines, and chemokines that regulate OA progression are anticipated. This will enable the development of specific immunotherapeutic strategies tailored to OA.

Although current research has preliminarily uncovered the critical role of immune cell differentiation in OA, many questions remain unresolved. For instance, the specific contributions of different immune cell subsets during various stages of OA are not yet fully understood; the dynamic regulatory mechanisms of immune cell differentiation and their interactions with the cartilage microenvironment require further exploration; additionally, the impact of individual differences (such as age, sex, metabolic status) on immune cell function warrants in-depth investigation.

In summary, targeting a single immune cell or cytokine for the treatment of osteoarthritis has certain limitations. To improve therapeutic efficacy and patients’ quality of life, it is essential to investigate the dynamic changes in immune cell infiltration, differentiation, and activation characteristics during different stages of OA progression and to develop more comprehensive treatment strategies based on these findings.

Therefore, elucidating the roles of different immune cells in cartilage damage and repair, as well as clarifying the mechanisms by which these immune cells influence OA cartilage damage and repair, not only helps to reveal the pathogenesis of the disease but also provides a critical foundation for developing personalized treatment plans, exploring immunomodulatory therapeutic strategies, and advancing the field of osteoarthritis treatment. Future research could leverage single-cell RNA sequencing technology, combined with genomics, epigenomics, metabolomics and proteomics data, to comprehensively uncover the interaction networks between immune cells and chondrocytes in OA, thereby developing immune cell-based regulatory therapies.
